# Clozapine‐associated severe eosinophilia following lithium rebound neutropenia: A case report

**DOI:** 10.1002/npr2.12143

**Published:** 2020-09-19

**Authors:** Kota Kikuchi, Norio Yasui‐Furukori, Saaya Yokoyama, Chie Hasegawa, Atsuhiko Kokubun, Shinichi Katsukura, Taro Shimizu, Kazutaka Shimoda

**Affiliations:** ^1^ Department of Psychiatry School of Medicine Dokkyo Medical University Tochigi Japan; ^2^ Department of Psychiatry Okamotodai Hospital Tochigi Japan; ^3^ Department of Diagnostic and Generalist Medicine School of Medicine Dokkyo Medical University Hospital Tochigi Japan

**Keywords:** clozapine, eosinophilia, lithium, neutropenia

## Abstract

**Background:**

Clozapine use is complicated by an increased risk of hematological adverse effects such as neutropenia and, rarely, eosinophilia.

**Case presentation:**

We present the case of a 48‐year‐old man with treatment‐resistant schizophrenia. On day 12 after clozapine initiation, he had a cough with a temperature of 39.8°C. On day 16, his leukocyte count had increased to 9320 cells/mm^3^ (neutrophils 7550 cells/mm^3^ and eosinophils 680 cells/mm^3^). We discontinued lithium because of neutrophilia and damage to renal function on day 20. His eosinophil count increased until day 29, reaching 6750 cells/mm^3^. We suspected a drug‐induced reaction and discontinued clozapine on day 30. His eosinophil count gradually decreased, reaching the normal range by day 40. However, his leukocyte and neutrophil counts also gradually decreased to below than the normal range by day 40. His leukocytes and neutrophil counts had recovered by day 55.

**Conclusion:**

We concluded that this patient had clozapine‐associated severe eosinophilia following lithium rebound neutropenia.

## Abstract 2

The eosinophil count increased reaching 6750 cells/mm^3^ with bronchitis and hepatitis during clozapine treatment. The leukocyte and neutrophil counts gradually decreased to below than the normal range by day 40 after discontinuation of lithium.

## Graphical abstract

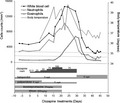

## 1 INTRODUCTION

A recent network meta‐analysis suggested that clozapine is superior to other antipsychotics,^1^ and several guidelines regarding pharmacotherapy for schizophrenia recommend clozapine as the best choice for treatment‐resistant schizophrenia.^2^, ^3^ However, its use is complicated by an increased risk of hematological adverse effects such as neutropenia^4^ and, rarely, eosinophilia.^5^ Eosinophilia develops as an immunologically mediated response in association with diverse processes, including allergic, neoplastic, and infectious diseases.^6^ We present the case of a 48‐year‐old man with treatment‐resistant schizophrenia and clozapine‐associated severe eosinophilia and then neutropenia secondary to lithium discontinuation. Written consent for publication of the case report was provided by the patient.

## 2 CASE REPORT

**Figure 1:**
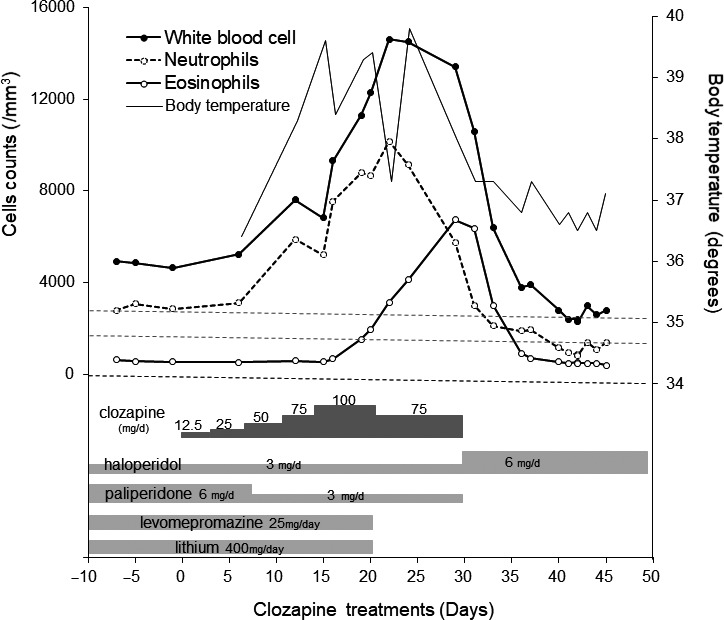
Clinical course of the patient including white blood cells, neutrophils, eosinophils, and body temperature during drug treatment. Upper and lower dotted lines indicate 3500 and 2000 cells/mm^3^, which is the lower limit for white blood cells and neutrophils, respectively, when clozapine is administered. The eosinophil count increased reaching 6750 cells/mm^3^ with bronchitis and hepatitis during clozapine treatment. The leukocyte and neutrophil counts gradually decreased to below than the normal range by day 40 after discontinuation of lithium

A 48‐year‐old male patient had been diagnosed with schizophrenia according to the DSM‐5 19 years prior. He had received 6 sessions of electroconvulsive therapy and several medications, such as haloperidol and risperidone, but the response was poor response. He was started on clozapine treatment (initially 12.5 mg/day, increased by 12.5 or 25 mg/d every 6‐7 days) because the schizophrenia was treatment resistant and his Positive and Negative Syndrome Scale (PANSS) score was 110. At the time of clozapine initiation, he received haloperidol 3 mg/d, paliperidone 6 mg/d, and levomepromazine 25 mg/d, which decreased in a cross‐titration regimen. He also received lithium 400 mg/d because his white blood cell (WBC) count was lower than 4000 cells/mm^3^ before clozapine. On day 12, he presented with a cough and a temperature of 39.8°C. By day 16, his leukocyte count had increased to 9320 cells/mm^3^ (neutrophils 7550 cells/mm^3^ and eosinophils 680 cells/mm^3^). The antibiotic ceftriaxone was administered, but it was not effective. On day 19, he was transferred to our hospital for detailed investigations. His PANSS score was 70. His admission laboratory tests showed the following results: WBC 11 300 cells/mm^3^ (3300‐8600 cells/mm^3^), neutrophil count 8780 cells/mm^3^ (1790‐6340 cells/mm^3^), eosinophil count 1540 cells/mm^3^ (0‐520 cells/mm^3^), hemoglobin (Hgb) 11.9 (13.7‐16.8 g/dL), platelet count 350 K/μL (158‐348 K/μL), aspartate aminotransferase 21 U/L (13‐30 U/L), alanine aminotransferase 91 U/L (10‐42 U/L), alkaline phosphatase 466 U/L (106‐322 U/L), gamma‐glutamyl transpeptidase 139 U/L (19‐64 U/L), creatinine 3.32 mg/dL (0.65‐1.07 mg/dL), estimated glomerular filtration rate 17.2 ml/min/1.73 m^2^ (>60.0 ml/min/1.73 m^2^), and C‐reactive protein (CRP) 16.9 mg/L (0.0‐0.14 mg/L). At admission, his medications were as follows: haloperidol 3 mg/day, paliperidone 6 mg/day, levomepromazine 25 mg/day, and lithium 400 mg/day. Imaging on admission included a chest X‐ray, which showed no abnormal shadows. A chest CT showed slight bilateral pleural effusion and pericardial effusion. We discontinued lithium because of neutrophilia and damage to renal function on day 20. We also switched from ceftriaxone to piperacillin/tazobactam on day 20 due to liver damage. CRP and creatinine peaked on day 20 at 17.2 mg/L and 3.39 mg/dL, respectively. WBCs, neutrophils, aspartate aminotransferase, alanine aminotransferase, and gamma‐glutamyl transpeptidase peaked on day 22, at 14 600, 10 160 cells/mm^3^, 79, 623, and 235 U/L, respectively (Figure 1). However, the eosinophil count continued to increase until day 29, reaching 6750 cells/mm^3^. We suspected a drug‐induced reaction and lowered the clozapine dosage to 75 mg/day starting on day 25, finally discontinuing clozapine on day 30 and increased haloperidol to 6 mg/d because of the increasing tendency toward eosinophilia. After that, the eosinophil count gradually decreased to within the normal range by Day 40. However, the leukocyte and neutrophil counts also gradually decreased to within the normal ranges, dropping below the normal ranges by Day 40 (Figure 1). We monitored the patient for 10 consecutive days based on suggestions from the clozapine patient monitoring service (CPMS). His leukocyte and neutrophil counts recovered by Day 55. His psychotic symptoms deteriorated and his PANSS score increased to 95 in spite of haloperidol at 6 mg/d on day 50, and no improvement was observed with aripiprazole at 30 mg/d and brexpiprazole at 2 mg/d on day 80. Retrospective analysis of the drug‐induced lymphocyte stimulation test (DLST) was positive for clozapine.

## 3 DISCUSSION

Here, we report the first case of clozapine‐associated severe eosinophilia and subsequent lithium discontinuation‐associated neutropenia. The patient also had bronchitis and hepatitis.

Clozapine eosinophilia has an incidence of 3% and mostly occurs in the first 2‐3 months after starting treatment. The largest study with 2,404 patients in Italy found an incidence of 2.2% based on the criteria of an eosinophil count greater than 400 cells/mm^3^.^7^ The most recent studies reported that approximately one‐tenth (N = 33; 9.9%) of patients developed eosinophilia (absolute eosinophil count >600 cells/mm^3^).^8^ In addition, cases of pancreatitis, hepatitis, colitis, nephritis, and myocarditis‐associated eosinophilia have been reported.^9^ Drug reaction with eosinophilia and systemic symptoms (DRESS syndrome) is an uncommon side effect of certain medications.^6^ The incidence is 0.4 cases per 1,000,000 in the general population, and the mortality rate is as high as 10%. In cases in which eosinophilia is accompanied by the involvement of major organs,^9^, ^10^ clozapine should be discontinued immediately. Because a rash was not observed, our patient did not meet the DRESS criteria, although other symptoms developed in our patient.

Several studies have suggested successful rechallenge after discontinuation of clozapine due to eosinophilia.^11^, ^12^ However, most cases included mild to moderate eosinophilia without organ damage. Our patient had severe eosinophilia with bronchitis and hepatitis, and he strongly refused a clozapine rechallenge.

Based on the clinical course of neutropenia after clozapine and lithium discontinuation, it appears that the patient had not only clozapine rebound neutropenia but also lithium rebound neutropenia. Because the WBC count before the initiation of clozapine was lower than 4000 cells/mm^3^, which is the Japanese criteria for the initiation of clozapine, lithium was added,^13^ and the WBC count increased to more than 4000 cells/mm^3^. Tomita et al reported that leukopenia and neutropenia appeared to develop in a patient owing to lithium discontinuation.^14^ There has been no report suggesting the existence of clozapine discontinuation‐associated neutropenia, although there was one case of rebound insomnia reported after abrupt clozapine withdrawal.^15^ Therefore, it is more likely that the lithium discontinuation was associated with the neutropenia in this case.

## CONFLICT OF INTEREST

Norio Yasui‐Furukori has been a speaker for Otsuka Pharmaceutical Co., Ltd., Mochida Pharmaceutical Co., Ltd., Dainippon Sumitomo Pharmaceutical Co., and MSD Co. Kazutaka Shimoda has received research support from Novartis Pharma KK, Dainippon Sumitomo Pharma Co., Astellas Pharma Inc, Meiji Seika Pharma Co., Ltd., Eisai Co., Ltd., Pfizer Inc, Otsuka Pharmaceutical Co., Ltd., Daiichi Sankyo Co., and Takeda Pharmaceutical Co., Ltd., and honoraria from Eisai Co., Ltd., Mitsubishi Tanabe Pharma Corporation, Takeda Pharmaceutical Co., Ltd., Meiji Seika Pharma Co., Ltd., Janssen Pharmaceutical KK, Shionogi & Co., Ltd., Dainippon Sumitomo Pharma Co., Daiichi Sankyo Co., and Pfizer Inc The funders did not have any role in data collection or in the study design, analysis, decision to publish, or preparation of the manuscript. The remaining authors declare that they have no competing interests to report.

## AUTHOR CONTRIBUTIONS

KK, NYF, SY, CH, AK, and SK were involved in the clinical investigations. NYF wrote the manuscript. TS, NYF, and KS were involved in the literature review and revisions. All authors read and approved the final manuscript.

## ETHICS APPROVAL AND CONSENT TO PARTICIPATE

The ethics committee of the School of Medicine at Dokkyo Medical University determined that there was no need to review this case.

## CONSENT FOR PUBLICATION

Written informed consent was obtained from the parent for the publication of this case report.

## Data Availability

Data sharing is not applicable to this article as no datasets were generated or analyzed during the current study.
